# Efficacy and safety of SH003 on immune function: study protocol for a randomized, double-blind placebo-controlled clinical trial

**DOI:** 10.1186/s12906-025-05032-4

**Published:** 2025-07-30

**Authors:** Chunhoo Cheon, Seong-Gyu Ko

**Affiliations:** Department of Preventive Medicine, College of Korean Medicine, Kyung Hee University, 26 Kyungheedae-ro, Dongdaemun-gu, Seoul, 02447 Republic of Korea

**Keywords:** SH003, Immune function, Natural killer cell activity, Randomized controlled trial, Traditional medicine, Astragalus Membranaceus, Angelica gigas Radix, Trichosanthis Radix

## Abstract

**Background:**

The immune system is essential for defending the body against pathogens and maintaining homeostasis. Recent global public health challenges have increased interest in natural products that enhance immune function. SH003, a compound derived from Astragalus membranaceus, Angelica gigas Radix, and Trichosanthis Radix, has shown potential as an immunomodulatory agent. This study aims to evaluate the efficacy and safety of SH003 in improving immune function in individuals with a mildly weakened immune systems.

**Methods:**

This randomized, double-blind, placebo-controlled trial will be conducted over 8 weeks with 120 participants aged 19 to 75 years who have experienced two or more upper respiratory infections in the past year. Participants will be randomly assigned to receive either SH003 or a placebo. The primary outcome is the change in natural killer cell activity. Secondary outcomes include cytokines levels (IL-2, IL-12, IFN-γ, TNF-α), inflammation markers (CRP, ESR), and stress and respiratory symptoms. Safety outcomes will be monitored through vital signs and laboratory tests.

**Discussion:**

This study represents the first clinical trial to evaluate the immune-enhancing effects of SH003 in a general population with mild immune system impairment. The results could offer valuable insights into the potential of SH003 as a novel immune support intervention.

**Trial registration:**

Clinical Research Information Service (registration number: KCT0009424, May 10, 2024, https://cris.nih.go.kr). Retrospectively registered.

## Background

Immunity, the defence system of body, is essential for recognizing and eliminating invading pathogens such as microbes, bacteria, viruses, as well as unnecessary internal tissues and by-products, thereby maintaining homeostasis [1]. Imbalances in this defence mechanism can lead to disease, highlighting the necessity for a well- regulated immune system to sustain health. Immune responses are broadly categorized into innate and adaptive immunity. The innate immune response, triggered within hours of pathogen entry, is mediated by host pattern recognition molecules and phagocytes equipped with Toll-like receptors [2]. In contrast, the adaptive immune response, though slower, strengthens immune reactions against the pathogen through memory response after initial defences are activated [3].

Recent environmental challenges, including fine dust and stressors, have heightened interest in immune-enhancing foods, particularly following public health crises like the MERS outbreak in 2015 and the COVID-19 pandemic [4]. Data from Statistic Korea in 2022 show that 46.5% of the population consumed health supplements primarily for disease prevention, with a significant portion planning to maintain or increase their intake [5]. The surge in health consciousness has spurred research into functional foods that enhance immune function and overall health.

Among the various natural compounds under study, Astragalus membranaceus is known in traditional medicine for its broad spectrum of health benefit, including its role in enhancing immunity and metabolic health [6–8]. Angelica Gigas Radix and Trichosanthis Radix are also notable for their medicinal properties, ranging from anti-inflammatory effects to enhancing metabolic and immune system functions [9–14].

SH003, a compound derived from these three plants, has been primarily studied for its anticancer properties with several preclinical study and clinical trials as an anticancer agent [15–17]. Recently, its potential to improve immune function was recognized following efficacy tests suggesting a new therapeutic avenue for SH003 in immunomodulation [18]. This study aims to conduct a randomized, double-blind, placebo-controlled trial to evaluate the effect of SH003 on human immune function, reflecting its potential as a novel immunostimulatory agent.

## Method/design

### Study design

This trial protocol (Version 1.1, approved on February 1, 2024) is designed as a single-center, randomized, double-blind clinical study to evaluate the efficacy and safety of SH003 in enhancing immune function over an 8-week period. This trial is a definitive, confirmatory study designed to evaluate clinical efficacy. The study has been approved by the Institutional Review Board of Jeonbuk National University Hospital in Republic of Korea and is structured and will be reported in accordance with Standard Protocol Items: Recommendations for Interventional Trials and Consolidated Standards of Reporting Trials guidelines [19, 20]. The study procedures are detailed in Table 1. Any important protocol modifications will be communicated to investigators during investigator meetings, and the final study design will be described in the resulting manuscript.

**Table 1:**
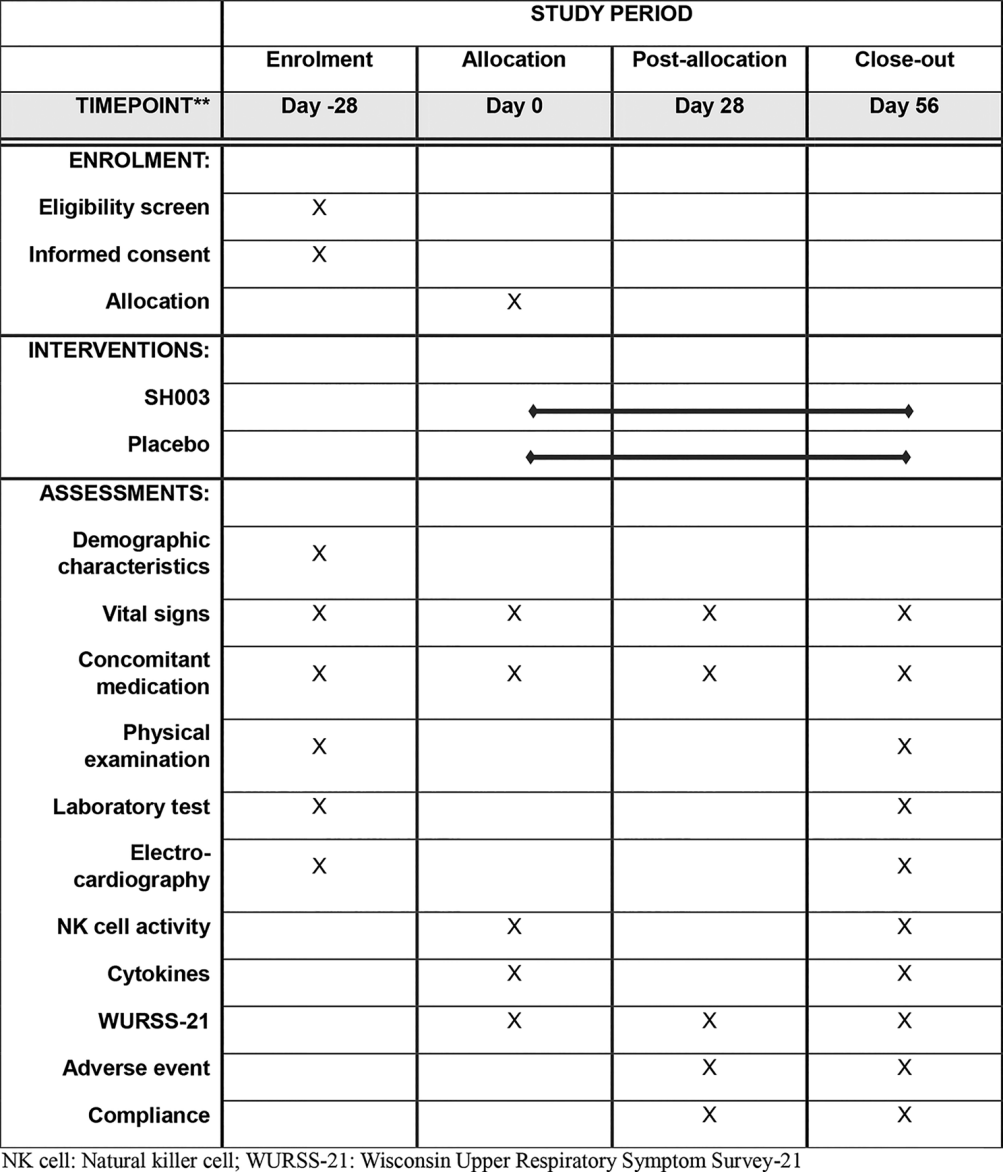
Study schedule

## Participants

### Recruitment

Participants will be recruited through advertisements posted on the bulletin boards and websites of the clinical trial institution, as well as on relevant organizational websites.

#### Inclusion criteria

1. Adults aged 19 to 75 years at the time of screening.
2. Individuals who have experienced two or more upper respiratory infections (such as the common cold, tonsillitis, pharyngitis, laryngitis, sinusitis, otitis media, and rhinitis) with in 12 months prior to screening.
3. Individuals who have been fully informed about the clinical trial and have provided written informed consent to participate.

#### Exclusion criteria

1. Individuals with a WBC cound below 3000/µL or above 8000/µL at screening.
2. Individuals who have received vaccinations (e.g., influenza, shingles, pneumococcal, COVID-19) within two months prior to the first intake of the investigational product.
3. Individuals with a body mass index below 18.5 kg/m^2^ or above 35 kg/m^2^ at screening.
4. Individuals taking hypertension medication.
5. Individuals with thyroid disorders.
6. Individuals undergoing treatment for or having uncontrolled diabetes (HbA1c > 7%).
7. Individuals requiring treatment for clinically significant acute or chronic conditions affecting the cardiovascular, endocrine, immune, respiratory, hepatobiliary, renal and urinary, neuropsychiatric, musculoskeletal, inflammatory, hematological, oncological, or gastrointestinal systems.
8. Individuals who have taken medications or health supplements related to immune function within one month prior to screening.
9. Individuals with a history of significant hypersensitivity reactions to any component of the ingredients.
10. Those who have undergone antipsychotic drug treatment within three months prior to screening.
11. Individuals with a history or suspicion of alcohol or substance abuse.
12. Those who have participated in another clinical trail within three months prior to screening.
13. Individuals whose diagnostic laboratory tests show AST and ALT levels exceeding three times the upper reference limit or serum creatinine levels greater than 2.0 mg/dL.
14. Pregnant of breastfeeding women.
15. Women of childbearing potential not agreeing to use adequate contraception.
16. Individuals deemed unsuitable for study participation by the principal investigator due to diagnostic laboratory results or other reasons.

### Randomization and allocation concealment

Eligible participants who meet the inclusion and exclusion criteria are randomly assigned to either the SH003 group or the placebo group. Randomization occurs after participants are confirmed eligible following their screening assessments. Participants are allocated in a 1:1 ratio to receive either the investigational drug or placebo, with the random sequence generated by the SAS 9.4 software for a total of 120 participants. An independent Contract Research Organization is responsible for generating the random allocation sequence. This sequence is then securely transmitted to the IP manufacturing facility. At this facility, each trial or placebo medication is produced according to the assigned random number and appropriately labeled to ensure blinding. Upon enrollment confirmation, participants are assigned their random numbers by the clinical research coordinator at the clinical trial site. This process ensures that both the participants and the clinical research team remain unaware of the allocation, thereby maintaining the integrity of the double-blinding method throughout the trial.

### Blinding

To maintain the integrity of the double-blind methodology in this study, the placebo used is indistinguishable from the IP in terms of shape, color, and scent. The labels for each unit of medication are applied at the manufacturing site before distribution to the clinical trial site. This procedure guarantees that neither the researcher nor the participants can identify whether a given treatment is the IP or the placebo, thus upholding the double-blinding throughout the duration of the trial. In the event of a serious adverse event that necessitates discussion regarding discontinuation of the study, unblinding may be permitted. In such cases, the unblinding procedure will be conducted through the contract research organization to ensure proper documentation and minimal bias. This will only occur when knowledge of the allocated intervention is essential for the safety of participant.

### Intervention

Participants will be randomly assigned to one of two groups upon their randomization, where they will receive a subject number. Over the course of 8 weeks, participants will orally ingest the investigational products twice daily following meals. SH003 group will take two tablets twice daily, once in the morning and once in the evening after meals, amounting to a total daily intake of 2 g of the active ingredient, SH003. Placebo group will also take two tablets twice daily, in the morning and evening after meals, but these capsules will not contain the active ingredient, amounting to a total daily intake of 0 g of SH003. During the trial period, the use of other medications or health supplements that may affect immune function will be prohibited.

### Investigational product and placebo production

The IP, designated as SH003, is composed of a 1:1:1 ratio of Astragalus membranaceus, Angelica gigas, and Trichosanthis Radix extracted with 30% ethanol. Each oblong, red tablet contains 500 mg of the active formulation. Excipients such as microcrystalline cellulose and lactose mixed powder are included in the composition. The placebo tablets are identically manufactured in oblong, red form using maltodextrin, microcrystalline cellulose, and lactose mixed powder, ensuring visual similarity to the IP in appearance. Both the IP and placebo are produced at Hanpoong Pharm & Foods Co Ltd, a facility compliant with Korea Good Manufacturing Practice standards.

### Medication compliance assessment

Medical compliance will be assessed during the third and fourth visits by examining the remaining quantities of the investigational products brought by participants. Participants are required to return any unused medication to the pharmacy, where it will be documented in the IP inventory and dispensing log. Compliance will be calculated using the following formula:

$$\eqalign{& \>{\rm{Compliance}}\>\left( \% \right) = \cr & \,\,\,\,\,\,\,\,\,\,\,\,\,\,\,{\matrix{{\rm{number}}\>{\rm{of}}\> \hfill \cr {\rm{products}}\>{\rm{consumed}} \hfill \cr} \over \matrix{{\rm{number}}\>{\rm{of}}\>{\rm{products}}\>{\rm{that}}\> \hfill \cr {\rm{were}}\>{\rm{supposed}}\>{\rm{to}}\>{\rm{be}}\>{\rm{consumed}} \hfill \cr} } \times \>100 \cr} $$

### Outcome measure

#### Demographic and baseline information

Demographic information includes sex, age, alcohol consumption, smoking habits, medical and medication history, physical examination, and vital signs (blood pressure, pulse rate, body temperature), Anthropometric measurements include height, weight and body mass index.

### Primary outcome

The primary outcome will involve the isolation of cells using a natural killer cell (NK cell) isolation kit on hepain-treated venous blood, followed by the measurement of NK cell activity. The NK cell activity will be measured using the lactate dehydrogenase assay, with K562 (a human leukemia cell line) as the target cells. Effector to target cell ratios of 2.5:1, 5:1, and 10:1 will be employed. NK cell activity will be assessed using the CytoTox 96 W Non-Radioactive Cytotoxicity Assay Kit (Promega Co., WI, USA).

### Secondary outcomes

Secondary outcomes will include cytokines (IL-2, IL-12, IFN-γ, TNF-α), inflammatory markers (CRP, ESR), patient global assessment, and stress levels as measured by the Global Assessment of Recent Stress Scale. Additionally, the Wisconsin Upper Respiratory Symptom Survey-21 (WURSS-21) will be utilized.

### Safety outcomes

Safety outcomes will be assessed through monitoring of vital signs, hematological tests, biochemical tests, and urinalysis to ensure safety throughout the study. If serious adverse event occurs, or if an adverse event arises that leads the participants or investigator to decide to discontinue the intervention, the clinical trial will be terminated for that participants. All participants experiencing an adverse event will be followed up until resolution or stabilization. Any loss or harm caused by this clinical trial will be covered by insurance reimbursement.

### Sample size calculation

The study aims to evaluate the immune function effects of SH003 intake over 8 weeks, compared to a placebo. Since no prior studies have investigated the immune-modulating effects of SH003, the sample size calculation is based on data derived from other studies on natural products with immune-enhancing effects. The sample size calculation was based on a NK cell activity change of 5.62% in the SH003 group and 0.95% in the placebo group, with a standard deviation of 8.24% in the SH003 group and 8.05% in the placebo group [21]. To achieve a statistical power of 80% (β = 0.2) at a 5% significance level (α = 0.05), with a two-tailed test and an effect size of 4.67%, the required sample size was calculated. Assuming an equal allocation ratio (1:1) between the SH003 and placebo groups, and accounting for a 20% dropout rate, a total of 120 participants (60 per group) will be recruited for the study.

### Statistical analysis

Descriptive statistics will be presented for the primary efficacy variable (NK cell activity) and secondary efficacy variables (cytokines, WURSS-21, inflammation markers, patient global assessment (PGA), global assessment of recent stress scale) for each group. Between-group comparisons will be conducted using independent t-tests on changes from baseline to week 8, while within-group comparisons will be analyzed using paired t-tests. For repeated measurements of WURSS-21, comparisons will be made using repeated measures ANOVA or linear mixed models. Post-hoc analysis will be conducted for variables showing significant differences between groups. For PGA at week 8, frequencies and percentages will be presented by group, and between-group differences in improvement will be analyzed using the chi-square test or Fisher’s exact test. If normality assumptions are violated, appropriate data transformations (e.g., logarithmic transformation) will be applied, or non-parametric tests such as the Wilcoxon signed-rank test and Mann-Whitney U test will be used.

Data obtained from participants in this clinical trial will be analyzed across four populations: Safety, ITT (Intent-to-treat), FA (Full Analysis), and PP (Per Protocol). The Safety population includes participants who took at least one dose of the investigational product. The ITT population includes all participants who were assigned a study number. The FA population consists of participants who were randomized and have at least one post-baseline efficacy evaluation. The PP population includes those in the FA group who completed the trial without major protocol violations. Efficacy evaluations will primarily use the PP population, supplemented by ITT and FA analyses. Safety evaluations will be conducted using the Safety population.

### Data collection and monitoring

Monitoring will be conducted to ensure the protection of the rights and welfare of study participants, and to verify that reported trial data are accurate, complete, and verifiable against source documents. The monitoring process will also confirm compliance with the approved study protocol, ICH-GCP guidelines, and applicable regulations. The monitor will perform regular on-site visits and telephone communications with the trial site. During these visits, the monitor will review essential documents, including original participant records, product management logs, and study files. They will also oversee progress of the trial and discuss any issues with the investigators. Monitoring will be conducted by an independent contract research organization. Data entry will be conducted using double data entry to minimize errors, and range checks will be implemented for data values to ensure accuracy. Data will be coded according to standardized procedures, and all electronic data will be securely stored with restricted access to authorized personnel only. There is no plan for an interim analysis.

## Discussion

This study represents the first clinical trial evaluating the immune-enhancing effects of SH003, a compound previously studied primarily for its anticancer properties and its efficacy in alleviating symptoms in cancer patients. The current trial broadens the application of SH003 by assessing its potential to enhance immune function in a general population. While the immune health market is dominated by a few well-established products, the findings of this trial could offer an additional option for consumers seeking immune support. Furthermore, this research could open new avenues for using SH003 in cancer patients by supporting their immune function from a different therapeutic angle.

SH003 was originally developed with a focus on its anticancer effects and was later evaluated for its ability to mitigate side effects of cancer therapies [22, 23]. Studies have also investigated the combination of SH003 with anticancer agents [17, 24], and its potential for enhancing immune function, particularly in stimulating NK cell activity, has been reported [18]. Based on this previous finding, this clinical trial was designed to be the first to target individuals with mildly weakened immune systems, rather than cancer patients. This shift in focus expands the potential therapeutic uses of SH003 significantly.

One limitation of this study is that the inclusion criteria, which focus on individuals who have experienced two or more upper respiratory infections in the past year, without relying on specific biomarkers, which may make it challenging to accurately assess efficacy. Additionally, as a single-center trial, the generalizability of the results may be limited. However, the study design adheres to the immune function evaluation guideline set by the Korean Ministry of Food and Drug Safety [25], which strengthens its methodological rigor.

If SH003 is confirmed to be effective in improving immune function through this trial, further studies should explore its application in patients with more severely compromised immune systems. Additionally, in-depth investigations into the underlying mechanisms by which SH003 modulates immune pathways would provide valuable insights. If proven both effective and safe, SH003 could be developed as a general health supplement, potentially contributing to the well-being of a broader population.

## Data Availability

No datasets were generated or analysed during the current study.

## Abbreviations

NK cell: Natural Killer cell
WURSS-21: Wisconsin Upper Respiratory Symptom Survey-21
PGA: Patient Global Assessment
ITT: Intent-to-treat
FA: Full Analysis
PP: Per Protocol
